# Supplementary figures and images for: Generation and utilization of a HEK-293T murine GM-CSF expressing cell line
Source: PLoS One. 2021 Apr 9;16(4):e0249117. doi: 10.1371/journal.pone.0249117 (PMC8034741; doi:10.1371/journal.pone.0249117)

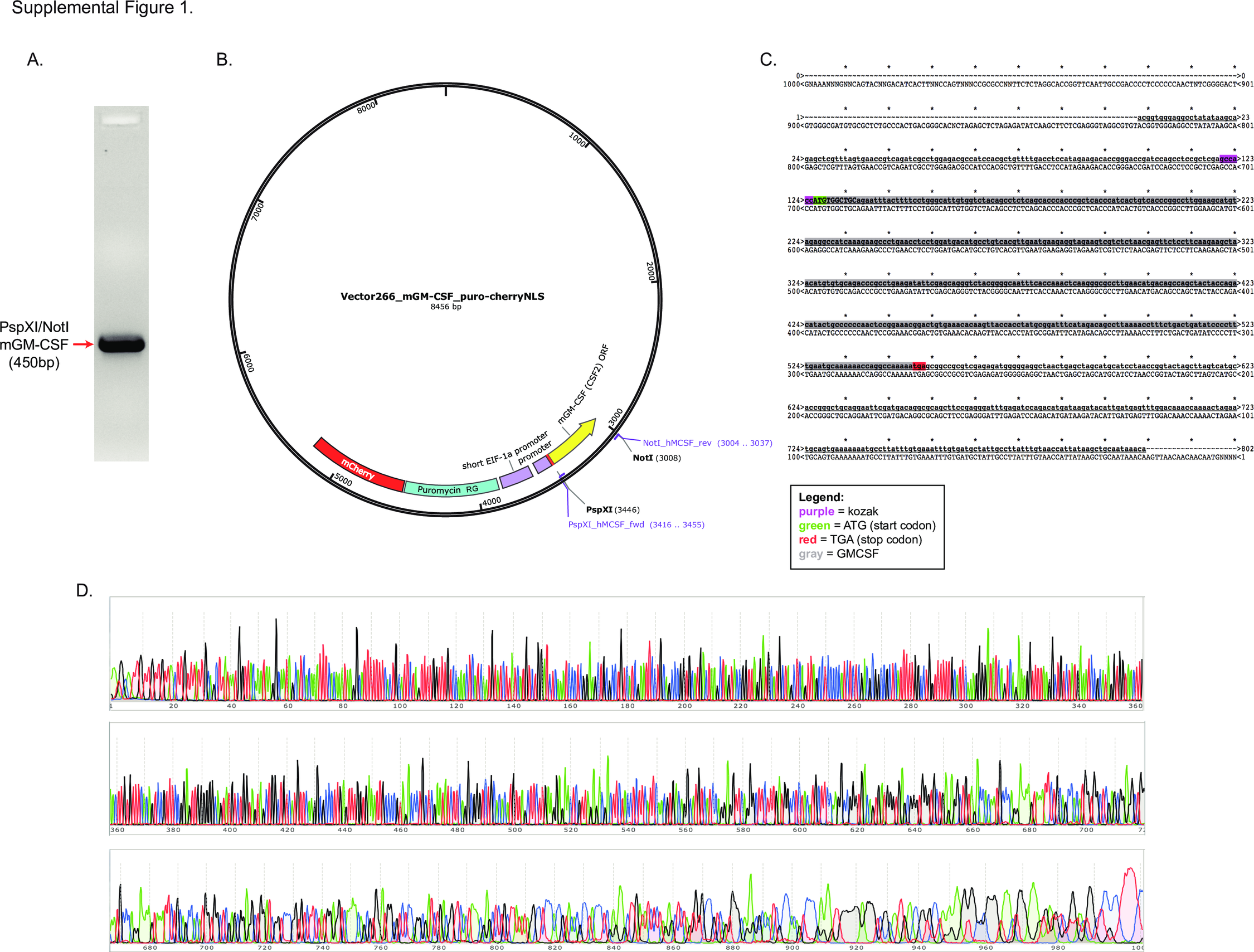

Supplement: S1 Fig — (A) Image of PspXI and NotI digested PCR product of murine GM-CSF. (B) Detailed 266 vector map of mGM-CSF, restriction sites, promoter, antibiotic selection and mCherry sequence. (C) Alignment of Sanger sequencing results of mGM-CSF and 266 Vector map strategy. (D) Quality of sanger sequencing results of murine GM-CSF. (TIF) [file pone.0249117.s001.tif]

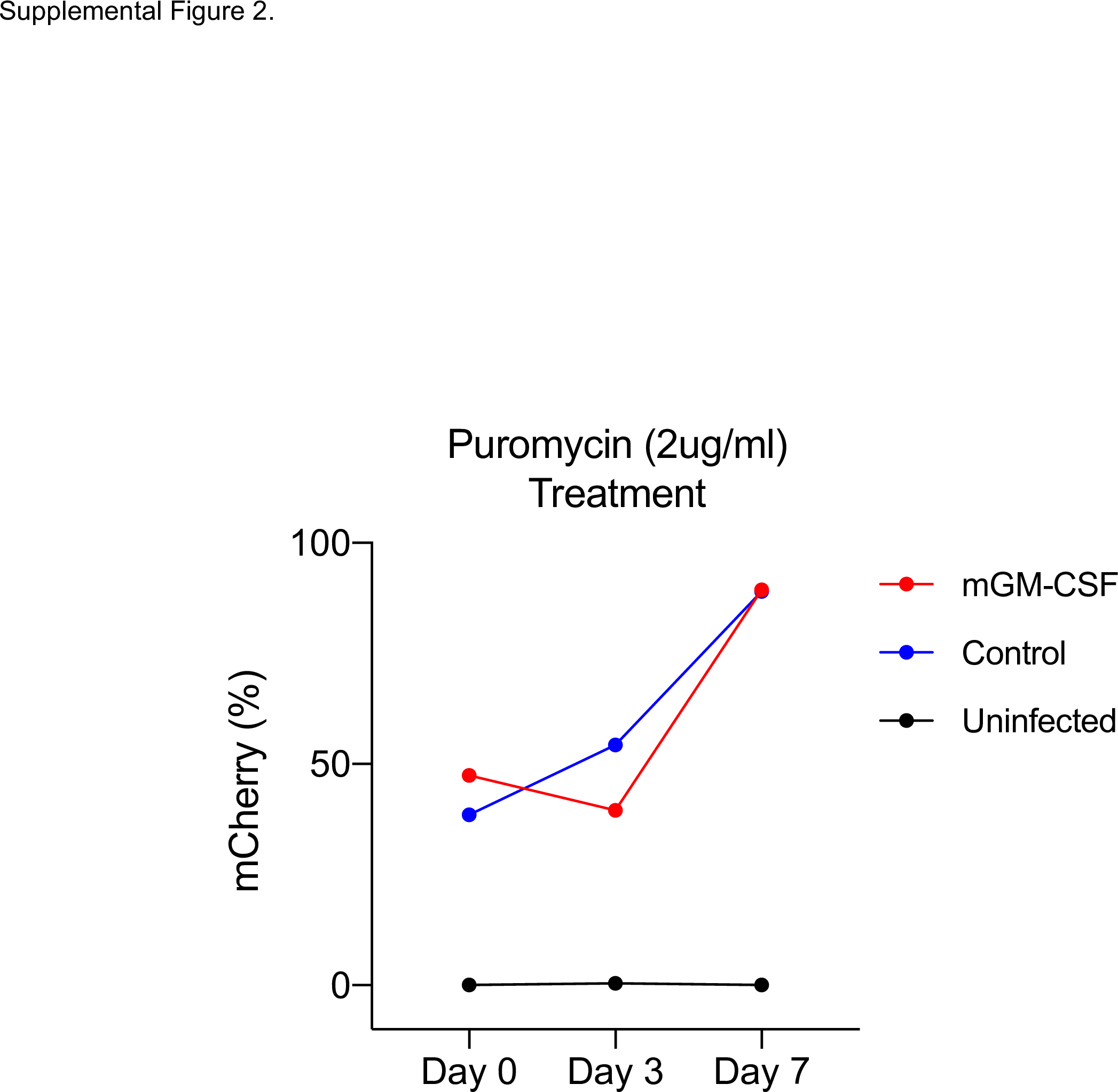

Supplement: S2 Fig — HEK-293T cell line lentiviral integration of GM-CSF was selected using puromycin (2 μg/ml) over a week. Flow cytometry was used to assess fluorescence intensity. (TIF) [file pone.0249117.s002.tif]

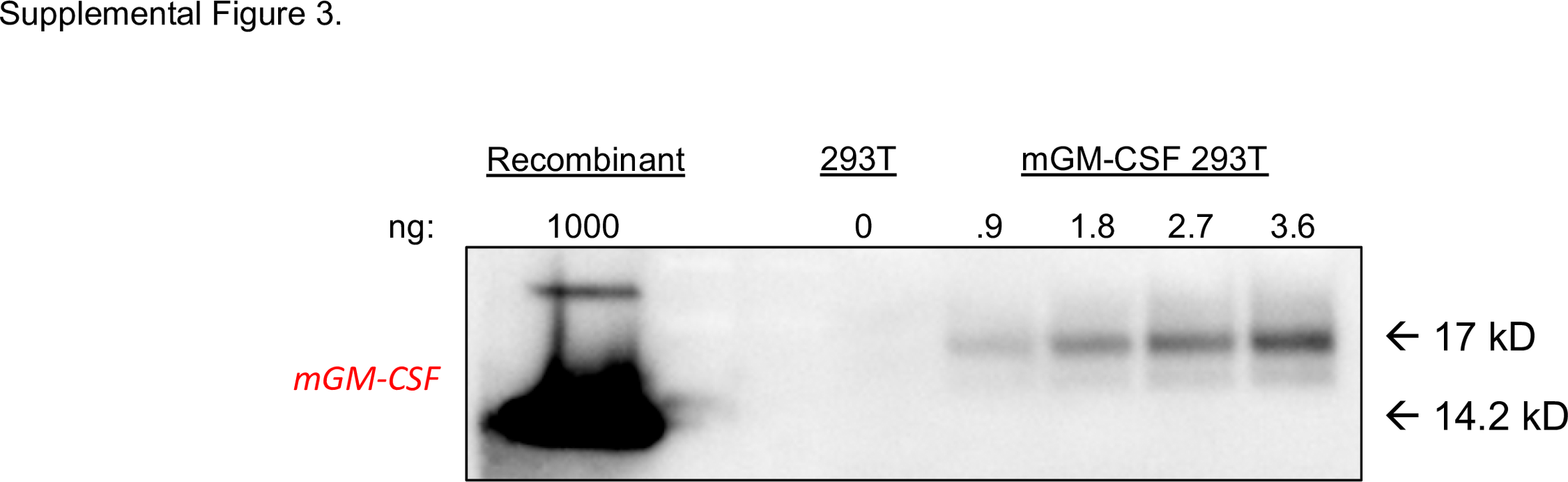

Supplement: S3 Fig — Western blot from left to right: 1 μg of pGM-CSF, Ladder, 20ul of 293T supernatant, 5 μl of supGM-CSF (0.9 ng), 10 μl (1.8 ng) of supGM-CSF, 15 μl (2.7 ng) of supGM-CSF and 20 μl (3.6 ng) of supGM-CSF. (TIF) [file pone.0249117.s003.tif]

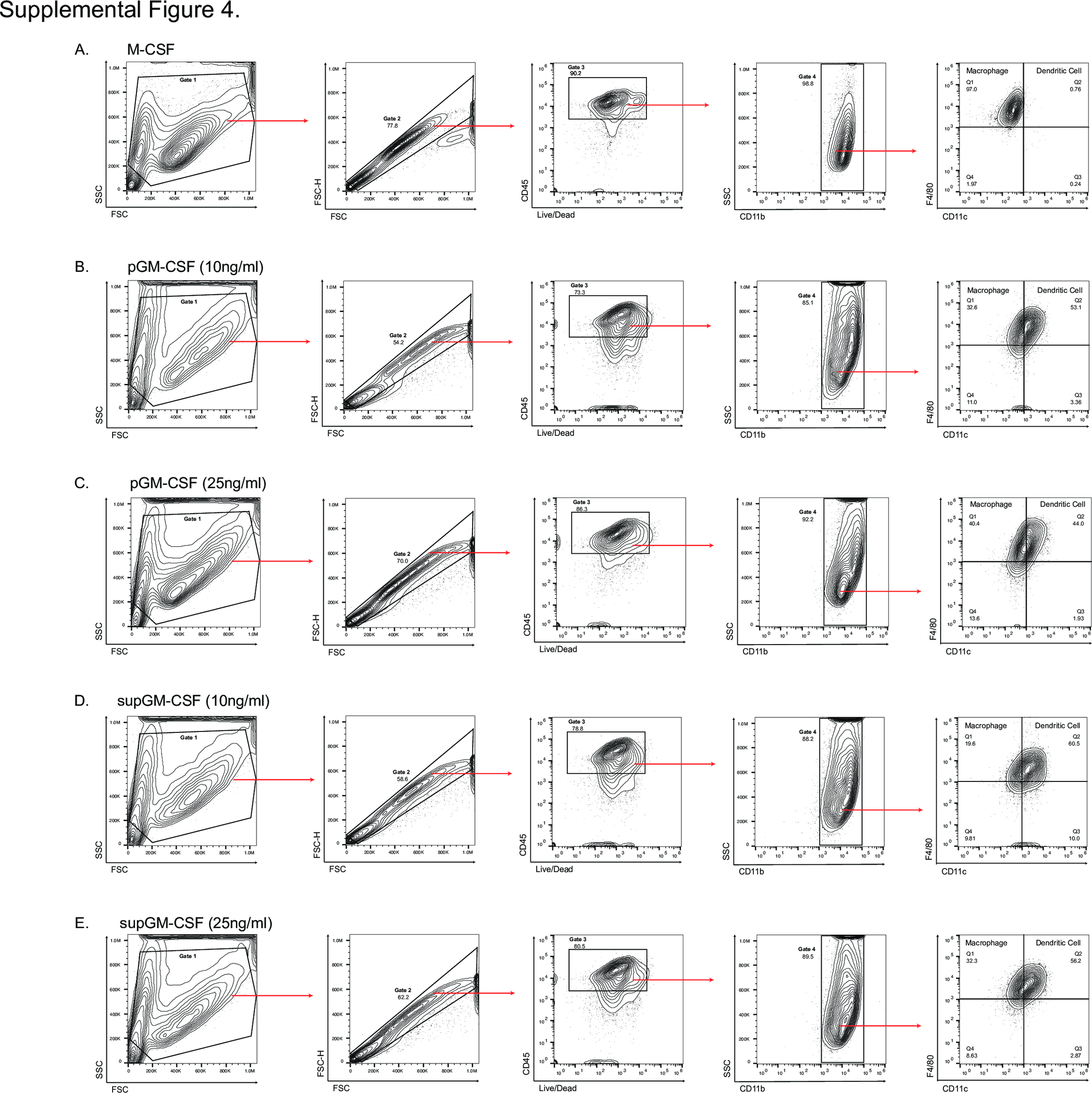

Supplement: S4 Fig — M-CSF and GM-CSF differentiated cells were both put through the same gating strategy. The 5 gating plots are for the (A) M-CSF, (B) pGM-CSF (10 ng/ml), (C) pGM-CSF (25 ng/ml), (D) supGM-CSF (10 ng/ml), (E) supGM-CSF (25 ng/ml) differentiated cells. Non-debris cells were gated for in gate 1, then singlets in gate 2, followed by CD45+ and live cells were gated for in gate 3, then CD11b+ cells were gated in gate 4 finally this population of cells were visualized using F4/80+ and CD11c+ markers and gate were put into quadrants. (TIF) [file pone.0249117.s004.tif]

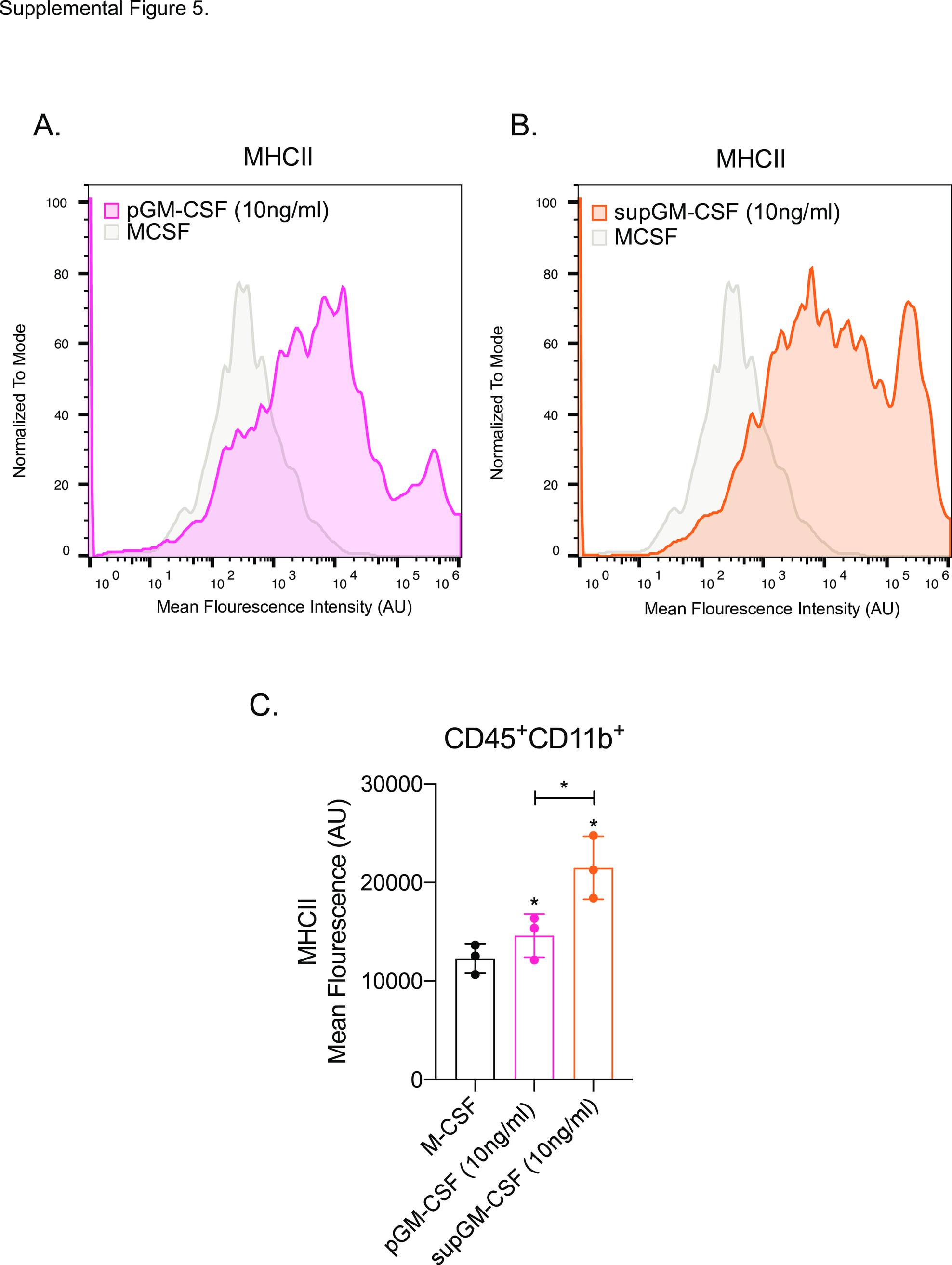

Supplement: S5 Fig — (A) Histogram overlay of CD45+ and CD11b+ M-CSF (grey) and pGM-CSF (pink) or (B) supGM-CSF (orange) differentiated cells expressing MHC II. (C) Graphical representation of MHC II mean fluorescence of M-CSF, pGM-CSF, or supGM-CSF differentiated cells. Student’s t-tests were performed using GraphPad Prism. Asterisks indicate statistically significant differences between mouse lines (*p ≥ 0.05, **p ≥ 0.01, ***p ≥ 0.005). (TIF) [file pone.0249117.s005.tif]

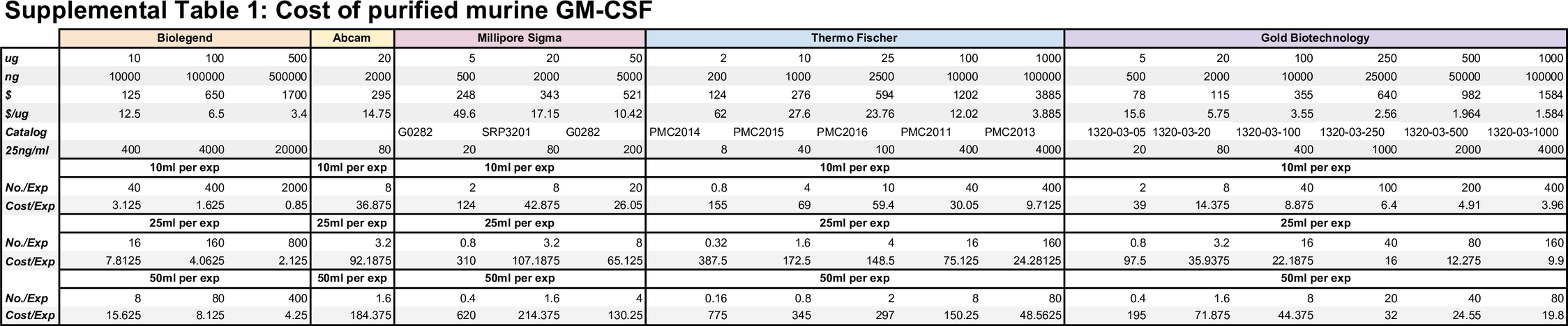

Supplement: S1 Table — (TIF) [file pone.0249117.s006.tif]

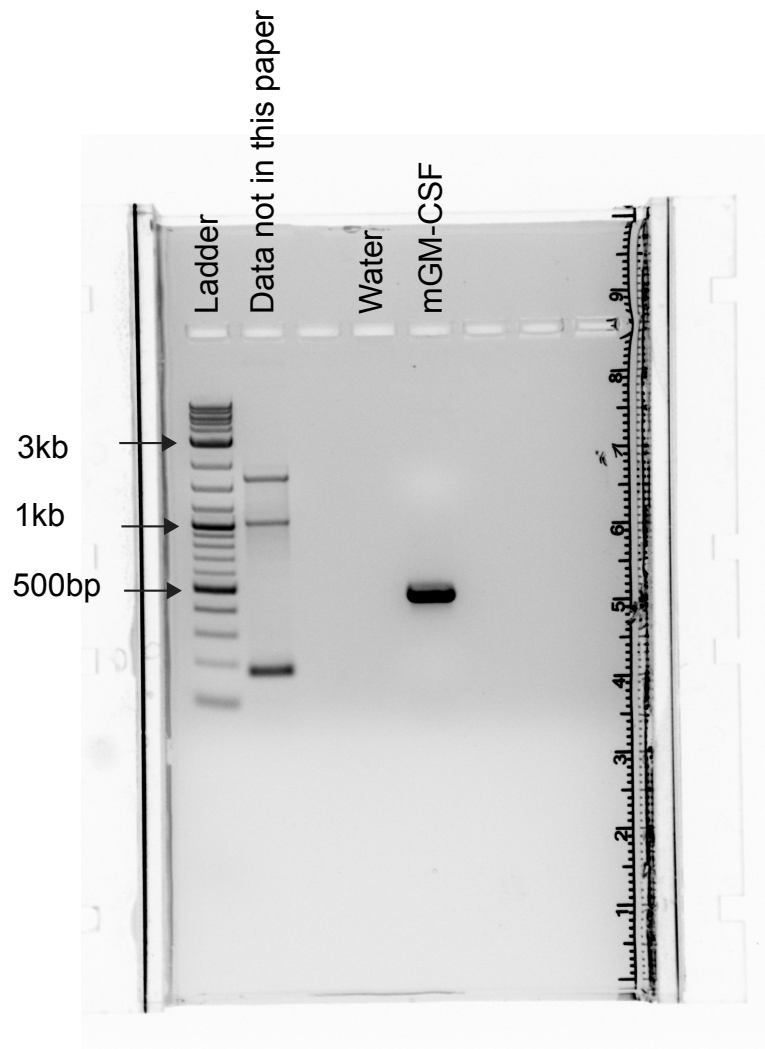

Supplemental Figure 3 Raw Data

| ng | <u>Recombinant</u> | <u>293T</u> |    | <u>mGM-CSF 293T</u> |     |     |
|----|--------------------|-------------|----|---------------------|-----|-----|
|    | 10000              | 0           | .9 | 1.8                 | 2.7 | 3.6 |

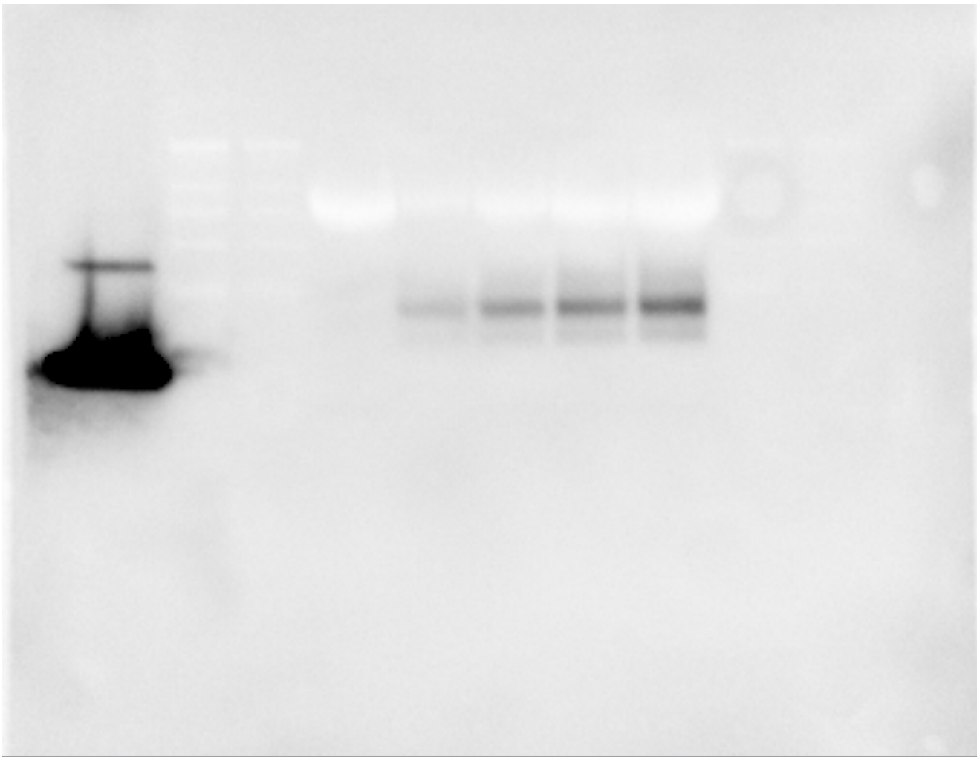

Supplement: S1 Raw Images — (PDF) [file pone.0249117.s009.pdf]
