# Supplementary material for: Generation and utilization of a HEK-293T murine GM-CSF expressing cell line
Source: PLoS One. 2021 Apr 9;16(4):e0249117. doi: 10.1371/journal.pone.0249117 (PMC8034741; doi:10.1371/journal.pone.0249117)
Supplement: S2 File — (DOCX) [file pone.0249117.s008.docx]

**S2 File. Extended methods**

Comprehensive Methods in the Creation of an mGM-CSF-Producing HEK293T Cell Line, Collection of mGM-CSF-rich Supernatant, and Using Cell Line Supernatant to Generate Dendritic Cells From Myeloid Progenitors

**Creating cell line**

1. Acquire mGM-CSF gene (Addgene: Plasmid #74465)
   1. Sequence to check for correct composition
2. PCR mGM-CSF gene
   1. Create and acquire primers with required restriction sites (NotI-HF and PspXI)
   2. PCR
3. Purify/isolate mGM-CSF gene (450 bp)
4. Ligation of mGM-CSF gene with pSico bidirectional vector plasmid
   1. Restriction digest of pSico bidirectional vector with Not1-HF and PspXI and purify
   2. Restriction digest modified mGM-CSF gene with Not1-HF and PspXI and purify
   3. Ligate vector with gene
5. Transform E. coli. with GM-CSF containing plasmid construct
6. Extract and isolate GM-CSF plasmid construct
   1. Incubate E. coli in liquid LB
   2. Miniprep
   3. Colony PCR and run through the gel to determine sample with GM-CSF gene inserted in pSico vector
7. Create lentiviral constructs containing plasmid
8. Infect HEK293 cells using lentiviral constructs
9. Select for cells with GM-CSF plasmid construct
   1. Allow cells to recuperate from infection and grow to confluence
   2. Puro-select
   3. FACS to determine concentration of mCherry+ cells (>90% required)

**Collecting mGM-CSF-rich supernatant**

1. Allow for HEK293 cells with plasmid construct to grow to confluency
2. (To be redone in T-175) Plate 2 million cells per 10 cm plate
3. Incubate for 3 days
4. Collect supernatant
5. If needed to confirm the concentration of mGM-CSF in the supernatant, perform ELISA (~200 ng/ml)

***In a T-175 flask**

1. Allow for HEK293 cells with plasmid construct to grow to confluency
2. Plate 9 million cells in 50 ml DMEM per T-175 flask
3. Incubate for 3 days
4. Collect supernatant
5. If needed to confirm the concentration of mGM-CSF in the supernatant, perform ELISA (~200 ng/ml)

**Generation of Bone-Marrow-Derived DCs**

*Day 0*

1. Sacrifice one or two mice and reserve femur and tibia of mice
2. Extract bone marrow cells from femur and tibia
3. In 6 well plate, plate equal amounts of cells into each well
   1. Total media should be equal to 2 ml per well (cells + DMEM + cytokine)
4. Incubate for three days

*Day 3*

1. On third day of differentiation, wash off dead cells
   1. Aspirate media
   2. Perform PBS wash
   3. Aspirate PBS
   4. Replace media

*Day 4 (if cells reach confluency)*

1. On fourth day of differentiation, move cells onto 10 cm plate
   1. Save conditioned media
   2. Perform PBS wash
   3. Aspirate PBS
   4. Using cell scraper, gently scrape cells off plate
   5. Transfer cells onto 10 cm plate
   6. Replace media
      1. Total media should be 10 ml (cells + DMEM + cytokine)
2. Incubate for 2-3 days

*Day 5 or 6*

1. Replace media
   1. Save conditioned media
   2. Perform PBS wash
   3. Aspirate PBS
   4. Replace media (10 ml total; 6.750 ml DMEM + 2 ml conditioned media + ~1.250 ml supernatant)
2. Incubate for 1-2 days

*Day 7 or until usage before day 14*

1. Replace media every 2-3 days
2. Cells finished differentiation. Ready for use.
