# Supplementary material for: Generation and utilization of a HEK-293T murine GM-CSF expressing cell line
Source: PLoS One. 2021 Apr 9;16(4):e0249117. doi: 10.1371/journal.pone.0249117 (PMC8034741; doi:10.1371/journal.pone.0249117)
Supplement: Attachment — Submitted filename: Response to Reviewers_mGMCSF_2020.pdf [file pone.0249117.s011.pdf]

PONE-D-20-18789 “Generation and utilization of a HEK-293T murine GM-CSF expressing cell line”

We would like to thank both of the reviewers for their helpful comments, suggestions and seeing the merit of this study. All changes to the text in the manuscript are completed and shown by track-changes. We believe we have addressed all the concerns as best we can and this has resulted in a much-improved manuscript. Below we outline our specific responses to each of the reviewer’s comments in blue.

***Editor Concise Comments***

The reviewers request a number of clarifications in the manuscript text, and *verification that the GM-CSF protein expressed from the transgene is of the expected size and was not fused to a cellular gene during the integration process.* To ensure rigor and reproducibility, please clarify whether the data presented represent multiple independent experiments or replicates from a single experiment. Please double-check the statistical analyses, for example the data presented in Fig. 3F do not appear to be significantly different but are marked as so. Please add statistical analyses to Fig. 4B-I.

1. Please ensure that your manuscript meets PLOS ONE's style requirements, including those for file naming. The PLOS ONE style templates can be found at

Thank you for this clarification. We have made sure that our manuscript meets your requirements.

2. Thank you for including your ethics statement: "In our work, we used mice for all experiments. These procedures were approved by IACUC, our number is CARPS1810.".

i) Please amend your current ethics statement to include the full name of the ethics committee that approved your specific study.

We have amended the ethics statement as advised it now reads:

All animal work was carried out in strict accordance with the recommendations in the Guide for the Care and Use of Laboratory Animals of the National Institutes of Health. The protocol was approved by the Institutional Animal Care and Use Committee at the University of California Santa Cruz (Protocol number CARPS1810).

ii) Once you have amended this/these statement(s) in the Methods section of the manuscript, please add the same text to the “Ethics Statement” field of the submission form (via “Edit Submission”).

The same text has now been added to the Ethics Statement

For additional information about PLOS ONE submissions requirements for ethics oversight of animal work, please refer to http://journals.plos.org/plosone/s/submission-guidelines#loc-animal-research

3. Thank you for stating the following financial disclosure: 'SCa received R21AR070973 funding for this work.'

At this time, please address the following queries:

a. Please clarify the sources of funding (financial or material support) for your study. List the grants or organizations that supported your study, including funding received from your institution.

Work on this project was supported by R21AR070973, an award from the National Institute of Arthritis and Musculoskeletal and Skin Diseases (NIAMS). Additional support was obtained from startup funds from UCSC to the Carpenter lab.

b. State what role the funders took in the study. If the funders had no role in your study, please state: "The funders had no role in study design, data collection and analysis, decision to publish, or preparation of the manuscript."

The funders had no role in study design, data collection and analysis, decision to publish, or preparation of the manuscript

c. If any authors received a salary from any of your funders, please state which authors and which funders.

Sergio Covarrubias received partial support from the R21AR070973 for his salary.

d. If you did not receive any funding for this study, please state: "The authors received no specific funding for this work."

**Please include your amended statements within your cover letter; we will change the online submission form on your behalf.**

*Reviewer #1*

1. As the authors are using a lentivirus to introduce the GM-CSF into the 293T cells. Do they know where the lentivirus integrated? Did they aim for a single integration or multiple integrations? If the lentivirus is integrated in multiple sites, this may affect the stability of the line.

We do not know where GMCSF is  integrated as it is random. We infected at a low MOI to try to ensure single integration but we have not confirmed this and since it is a heterogeneous line and not a clone there could be differences in different cells. Overall the line appears very stable as we have been working with it for a couple of years now and performed freeze thaws and continue to obtain consistent concentrations of mGM-CSF produced by elisa (Figure 1C).

2. The authors should run a western blot showing that GM-CSF is at the proper molecular weight. This is particularly important as they transduced the 293T cells with a lentivirus. ELISAs do not tell you if the secreted GM-CSF is intact or fused with a protein at the site of the lentiviral integration. Even if it is functionally active, showing that the GM-CSF secreted is at the right molecular weight and not fused with some cellular protein is of importance.

We would like to thank the reviewer for this useful suggestion. We have successfully performed a western blot for pGM-CSF and our supGM-CSF using the primary mGM-CSF antibody from R&D DY415-05. We obtained the purified GM-CSF from biolegend (Cat#576302), which is stated to be 14.2kD in the datasheet. We ran increasing concentrations of our undiluted supernatant from our mGM-CSF 293T cell line. We can clearly visualize our supGM-CSF as a single band which runs a little higher (~17kD) than the purified GM-CSF. The molecular weight of GM-CSF is 16kD therefore our band is closer to the correct size. It is possible that biolegend generated a slightly shorter version of the protein or that GMCSF generated in our cell line contains modifications that increase its molecular weight that are not observed in the pGM-CSF that was generated in E.Coli. We have now included this western blot as supplemental figure 3 and added additional discussion on this point lines 292-296.

Supplemental Figure 3

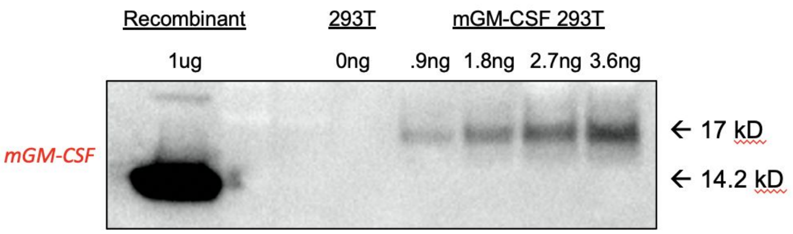


**Supplemental Figure 3: Size quantification of pGM-CSF and supGM-CSF.** Western blot from left to right: 1 mg of pGM-CSF, Ladder, 20ul of 293T supernatant, 5 ml of supGM-CSF (0.9 ng), 10 ml (1.8 ng) of supGM-CSF, 15 ml (2.7 ng) of supGM-CSF and 20 ml (3.6 ng) of supGM-CSF.

3. Line 102 “the following …mice” makes no sense. Please fix this part of the sentence

Many thanks for this comment, this has since been edited to “Commercial ELISAs were used to measure the following proteins in mice in triplicate”.

4. It is not clear in the figure legends or the text if the repeats of the BMDCs/BMDMs experiments (staining, response to LPS etc) were done using cells differentiated from one mouse or each repeat is BMDCs/DMs derived from a different mouse every time (i.e. cells from different mice are used for different repeats of the experiments). Please clarify.

We thank you for bringing up this important point. Our apologies for not making this clear. All experiments were done in biological triplicate, using different mice. This has now been clarified in all necessary figure legends.

5. In line 178 you say you use 10WT mice, but Figure 4A says 40. Which one is right?

Thank you for catching this typo, it has now been corrected in the manuscript. The correct number of WT is 40.

***Reviewer #2***

Line 80: "…depleted of erythrocytes." How was this done? Lysis buffer or other method?

Thank you for the attention to detail! The process in which we depleted erythrocytes was by using the Red blood cell lysis buffer (RBC) lysis buffer, specifically catalogue number 786-649 from biosciences.

Line 81-82: "…supplemented with either 10% M-CSF from L929 cells, 5-10% supernatant, or 10-25ng/ml of recombinant mGM-CSF" To what cells does "5-10% supernatant" refer? I presume it is the 293T cells, but this should be clarified here.

You are correct, we are implying our 293T cell line. We have now made this clearer. Changing the sentence to "10% M-CSF from L929 cells, 5-10% supernatant from the mGM-CSF 293T cell line, or 10-25 ng/ml of recombinant mGM-CSF [PMC2015, Thermo Fisher]".

Line 97: I suggest removing or replacing "inflammasome" in this method title because the activation of these cells includes LPS stimulation and does not appear to be specific to the inflammasome.

In this case, we maintain that the methods section should still be titled inflammasome activation because inorder to activate this pathway it requires two signals. The first signal is the LPS for priming, followed by the second signal Poly(dA:dT). Poly(dA:dT) is the ligand for the pattern recognition receptor (PRR), Aim2, which will subsequently form the inflammasome complex. This point was also made clearer in the results section lines 203-206.

Line 117: add citation for "Chen et al. (PMID:3288696)" to references

****Lets fix**

Line 153: "…CD11c is a dendritic cell marker" CD11c is also expressed by some macrophages.

Thank you for this point. We have now changed the sentence to: "Percent of cells in quadrant 2 (F4/80+ and CD11c+) was significantly higher for all GM-CSF treated cells, in comparison to M-CSF treated cells, as is expected since BMDMs express very little CD11c while BMDCs highly express CD11c."

Line 164-165: "Dendritic cells differentiated with 10ng/ml GM-CSF show statistically higher mean fluorescence intensity for MHC class II for supGM-CSF in comparison to pGM-CSF". The authors should cite Helft et al. (PMID: 26084029), which show that MHCII high population are more DC-like and the MHC-intermediate population is more macrophage-like. The differences noted by the authors in their

comparisons may relate to these two distinct populations reported by Helft et al. to be present within the GM-CSF cultures.

Thank you for this point, we have now included this finding in our discussion, along with the citation of the work from Helt et al.

Line 165-167: Although there is no difference in MFI overall, there appears to be a difference in proportion of MHCII-low/int cells between Fig. 3A and Fig. 3B.

We appreciate this observation and indeed, while the overall MFI between pGM-CSF and supGM-CSF is similar, the supGM-CSF does appear to have a more pure population and pGM-CSF has two distinct populations. We have now appended our results to include this point!

Line 221: "…while this pathway is now contested in DCs our results for GM-CSF is true" The meaning of this statement is unclear.

Thank you for addressing this confusion in the text. We have now reworded the sentence to be more direct. "..While there has been published reports that it is macrophages within the population of cells that arise from the GM-CSF model of differentiation and not the DCs that are responsible for the inflammasome activation we can show here that our $CD11c^{+}/MHC^{hi}$ DC cells possess a functional AIM2 inflammasome [29]."

Line 372: This extended method does not appear to be finalized. For example, there are ?'s remaining in the method text (line 381, 426, 436)

This is an error on our end, thank you so much for catching these typos.
