# Supplementary material for: Generation and utilization of a HEK-293T murine GM-CSF expressing cell line
Source: PLoS One. 2021 Apr 9;16(4):e0249117. doi: 10.1371/journal.pone.0249117 (PMC8034741; doi:10.1371/journal.pone.0249117)
Supplement: Attachment — Submitted filename: Response to Reviewers.pdf [file pone.0249117.s012.pdf]

PONE-D-20-18789 “Generation and utilization of a HEK-293T murine GM-CSF expressing cell line”

Dear Editor,

We appreciate your time and continued interest in our manuscript. We believe we have addressed all the concerns to the best of our abilities. Below we outline our specific responses to the four main comments/concerns and kept the quoted submission guidelines in red. Overall we continue to believe that this work has the scientific merit to be published in PLOS One.

Editor Comments

Submission guidelines: "Utility: The tool must be of use to the community and must present a proven advantage over existing alternatives, where applicable."

Submission guidelines: "Validation: If similar options already exist, the submitted manuscript must demonstrate that the new tool is an improvement over existing options in some way. This requirement may be met by including a proof-of-principle experiment or analysis; if this is not possible, a discussion of the possible application and some preliminary analysis may be sufficient".

1. **Conditioned media from your new cell line is more cost-effective than commercially available rGM-CSF. However, an alternative cell line, J558L-GM-CSF, is available and still in use today for the purposes described in your study, despite the 1997 study that indicated possible detrimental secretion of IL-10. Such studies include:**

Our tool, HEK-293T mGM-CSF cells is much more advantageous than the existing alternatives. When assessing the main alternative, recombinant GM-CSF, our cell line is much more economical as well as more efficient (Fig. 2C). The recombinant GM-CSF is the gold standard when it comes to both culturing bone marrow-derived dendritic cells (BMDCs) (https://doi.org/10.1002/cpim.115) and alveolar macrophages (10.1084/jem.20131199, 10.1189/jlb.1107781, 10.1007/978-1-4939-6625-7_23). While J558L, a myeloma, mGM-CSF cell line exists and was created in 1988 (https://doi.org/10.1002/eji.1830180115), it has not replaced recombinant mGM-CSF when it comes to general *in vitro* studies. This is primarily due to the fact that the cell line is derived from a lymphoid (immune cell) and therefore can secrete more than just mGM-CSF into the supernatant from this cell line, including the incredibly important anti-inflammatory cytokine IL10 found in the 1997 study (10.1002/eji.1830270326). When this supernatant is then cultured with bone marrow to differentiate into BMDCs or fetal livers to differentiate alveolar macrophages, for instance, it will affect the transcriptional programming (immune signaling) leading to a switch of a pro-inflammatory to an anti-inflammatory state. Our cell line, HEK-293T mGM-CSF is much more advantageous because these cells are considered “empty” in that they do not express these immune chemokines and will not alter the state of immune cells.

From publically available RNAseq datasets (https://www.proteinatlas.org/) [image below], we know that IL10 is not expressed in HEK-293T cell lines. This, in addition to how the cell line was generated and the cell type, makes our HEK-293T mGM-CSF cell line superior to the J558L mGM-CSF lymphoid expressing cell line.

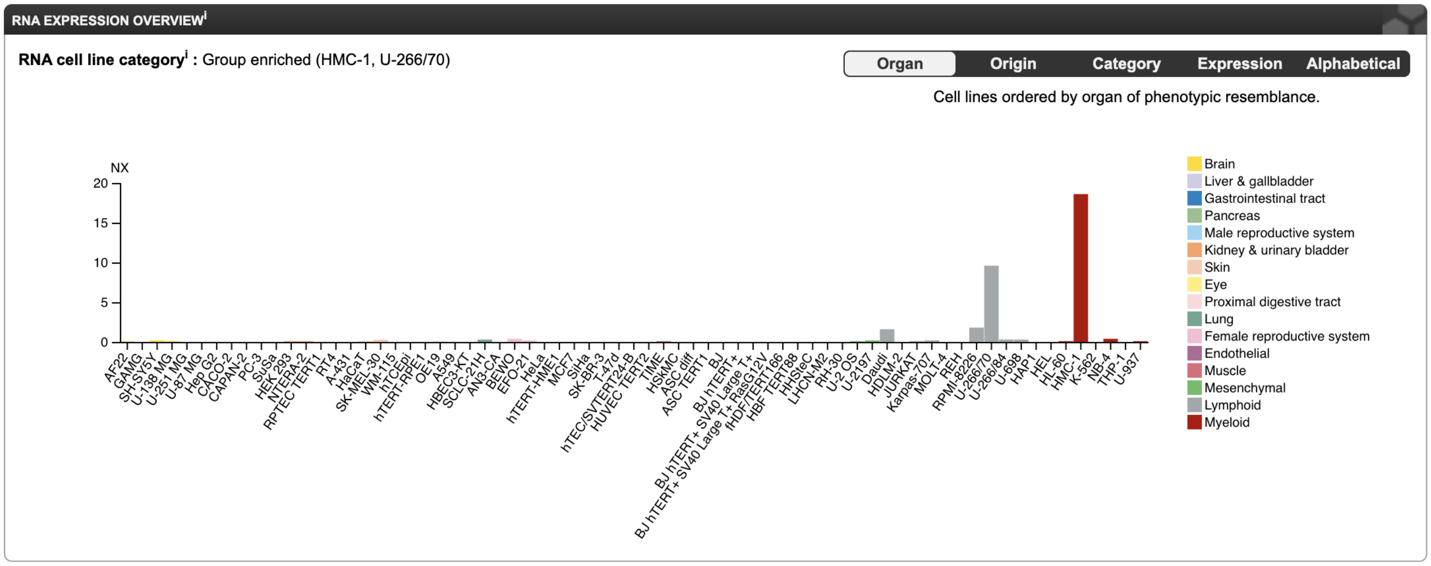

At this moment, the only additional cytokine that is secreted in J558L and not HEK-293Ts is IL10. IL10 is a heavily studied cytokine, defined as a potent anti-inflammatory that plays a central role in limiting host immune response to pathogens, thereby preventing damage to the host and maintaining normal tissue homeostasis (PMID: 22428854). In DCs a study from the Gregori group showed that IL10 can lead to a very specific subset of DCs, coined DC-10 in humans (10.3389/fimmu.2018.00682). Another study, by the Staege group shows that IL10 changes both the phenotype and gene expression of BMDCs (PMID: 24222115).

Additionally, papers that were indicated as sources that use J558L can be misleading. This is because J558, originally generated in 1972 (10.1073/pnas.80.3.825), was further subcultured and a J558L cloned was identified and defined as a spontaneous heavy chain-loss-variant myeloma cell line and does not express GM-CSF (10.1073/pnas.80.3.825).

Now, we would now like to discuss the papers you cited. First, the Cell study by the Colonna group shows the use of J558L specifically as a cancer cell line (https://doi.org/10.1016/j.cell.2017.11.037). In this study, J558L was utilized as a cancer cell line to show the anti-cancer properties of NK cells and PDGF-DD with the presence of pro-inflammatory cytokines (TNFa or IFNg). This study is utilizing J558L which does not express GM-CSF, which again is being primarily used as a myeloma lymphoid cancer cell line to determine novel anticancer mechanisms. The cell line was not utilized to generate Alveolar Macrophages or Dendritic Cells. Therefore this use does not discount the value of our HEK-293T mGM-CSF cell line. Second, the Cell Host & Microbe study by the Nice group does utilize J588L mGM-CSF to generate DCs (https://doi.org/10.1016/j.chom.2018.10.003). In this study the entirety of the study was not *in vitro*, however, I would be very concerned about how a potent anti-inflammatory protein would affect the cytokine profile of a primary BMDC. This would be a huge caveat to the experiment. We understand the costliness of rGM-CSF and this may outweigh the experiment caveats if the proper controls are used. However, we believe that our tool would be greatly advantageous to all researchers performing *in vitro* studies on BMDCs and Alveolar Macrophages with an interest in pro-inflammatory signaling. The final paper that was cited by you the editor was a 2019 study, our reference 19, showing the use of J558L mGM-CSF to culture alveolar macrophages (10.21769/BioProtoc.3302) from bronchiolar lavage (BAL) fluid. This protocol's main aim is to determine the best way to harvest the highest number of alveolar macrophages by assessing temperature and FCS or EDTA concentration in the media used to perform BALs. The cultured primary alveolar macrophages were then

counted, the activity of these cells was never measured. Thus the caveat of the IL-10 expression was not considered as critical and J558L was used instead of the recombinant GM-CSF standard. Therefore in conclusion we believe our tool is worth publishing in PLOS One and available as the new gold standard that will be a cheaper and overall more efficient alternative to rGM-CSF and does not come with the caveats of inflammatory cytokines as are associated with the J558L.

2. **Do you have experimental evidence that indicates that your cell lines produce conditioned media that is superior to that produced by the J558L cell line for the purpose of culturing BMDCs or alveolar macrophages? Is there evidence in the literature that J558L cell supernatant is problematic for the in vitro differentiation and culturing of the cell types used in your study?**

We were primarily interested in making sure that our cell line was more advantageous when compared to the gold-standard of BMDC or Alveolar Macrophage *in vitro* work. We chose to focus on making the comparison of our line to the most utilized alternative in the field with his recombinant GM-CSF. Given the caveats outlined above with the J558 line producing cytokines like IL10, we did not consider it to be the best comparison to make as it is will be clear to immunologists interested in studying signaling that J558L is just not an option for use in such studies. We also already know that the J588L cell line expresses immune proteins, including the anti-inflammatory protein IL10 (10.1002/eji.1830270326).

Submission guidelines: "Availability: If the primary focus of a manuscript is the presentation of a new tool... it should be openly available under a license no more restrictive than CC BY."

3. **Will your 293T-GM-CSF cell line be made available to other investigators? If so, please include this information in the Data and Materials Availability section (lines 303-306).**

This is a very important point. We would like to share that this cell line is available upon request. Since being published in bioRxiv, July 2020, our cell line has been sent to 4 separate labs for both culturing BMDCs and primary Alveolar Macrophages. The 'Data and Materials Availability' section has been amended (lines 305-306).

Submission guidelines: "Cell lines: For established cell lines, the Methods section should include: ... The cell line repository or company the cell line was obtained from, the catalogue number, and whether the cell line was obtained directly from the repository/company or from another laboratory."

4. **Please include this information in the Methods section. It will be particularly important for a reagent that may be used by other laboratories in the future.**

Material and Methods amended for HEK-293T cell line, line 70.
