# Supplementary material for: Generation and utilization of a HEK-293T murine GM-CSF expressing cell line
Source: PLoS One. 2021 Apr 9;16(4):e0249117. doi: 10.1371/journal.pone.0249117 (PMC8034741; doi:10.1371/journal.pone.0249117)
Supplement: Attachment — Submitted filename: Robinson etal.docx [file pone.0249117.s010.docx]

In this manuscript, Robinson et al describe a new cell line that they have developed that expresses constitutively GM-CSF. This 293T derived cell line that expresses high levels of GM-CSF in the media, is a cheap and efficient alternate source of GM-CSF that is usually bought commercially at high cost. They provide an array of experiments that show their supGM-CSF is as potent in the differentiation of BMDCs and AMs as the commercially acquired GM-CSF. Furthermore, they show that the BMDCs and AMs developed show no changes in their immune signaling cascades. Overall, this is a very interesting study and this cell line could make the study of BMDCs and AMs more affordable and easier to do.

Points

1. As the authors are using a lentivirus to introduce the GM-CSF into the 293T cells. Do they know where the lentivirus integrated? Did they aim for a single integration or multiple integrations? If the lentivirus integrated in multiple sites, this may affect the stability of the line.
2. The authors should run a western blot showing that GM-CSF is at the proper molecular weight. This is particularly important as they transduced the 293T cells with a lentivirus. ELISAs do not tell you if the secreted GM-CSF is intact or fused with a protein at the site of the lentiviral intergration. Even if it is functionally active, showing that the GM-CSF secreted is at the right molecular weight and not fused with some cellular protein is of importance.
3. Line 102 “the following …mice” makes no sense. Please fix this part of the sentence
4. It is not clear in the figure legends or the text if the repeats of the BMDCs/BMDMs experiments (staining, response to LPS etc) were done using cells differentiated from one mouse or each repeat is BMDCs/DMs derived from a different mouse every time (i.e. cells from different mice are used for different repeats of the experiments). Please clarify.
5. In line 178 you say you use 10WT mice, but Figure 4A says 40. Which one is right?
